# College Commencements

**Published:** 1897-04

**Authors:** 


### COLLEGE COMMENCEMENTS.

The Fortieth Annual Commencement Exercises of the Atlanta Medical College were held on the evening of March 31st, at the Grand Opera House. In spite of inclement weather the exercises attracted a large audience.

After the opening prayer by Dr. I. S. Hopkins, the Dean of the College, Dr. W. S. Kendrick, made his annual report. He was pleased to report the continued success of the College, and the further addition of necessary facilities for teaching. Thirty-one graduates were presented for the degree doctor of medicine, and seven graduates in pharmacy. These appeared in the conventional cap and gown, and Colonel N. J. Hammond, President of the Board of Trustees, addressed them in Latin and presented to each his diploma. Dr. T. P. Bullard, class valedictorian, delivered a polished and thoughtful oration.

The prizes were awarded as follows: First honor, Dr. C. W. Strickler; Second honor, Dr. J. F. Williams; Third honor, Drs. T. P. Bullard and J. R. Wells. After a short talk to the class the prizes were awarded by Professor C. C. Cox, of College Park. The following received honorable mention: Drs. J. A. Alley, T. E. Cunningham, J. S. Rogers, and P. L. Moon.

The closing address to the graduating class was delivered by Rev. Sam Jones, who made one of his characteristic talks in his own good-natured and inimitable style.

The Eighteenth Annual Commencement of the Southern Medical College was held in the Grand Opera House, March 30th. The exercises were opened with prayer by Bishop Cleland K. Nelson, after which Dr. J. B. Baird, Dean of the College, made his annual report. The College had just closed a very prosperous session, ninety-six students having been enrolled in the medical department alone, of which thirty composed the graduating class. Diplomas were then awarded the graduates by Hon. Howard Van Epps, President of the Board of Trustees. Mr. Lucien L. Knight delivered the Annual Address, in his usual felicitous and eloquent manner.

Dr. S. A. Visanska, Dean of the Pharmaceutical Department, made his report, and presented one candidate for the degree, Graduate in Pharmacy.

The first honor medal was won by Dr. M. J. White, of Georgia; the second by Dr. W. H. Moncrief, of Georgia; and the third by Dr. J. C. Haskell, of South Carolina. Drs. E. F. Wyatt, C. C. Fletcher, R. T. Dorsey, Jr., William Owens, and V. O. Harvard received honorable mention.

The Opera House was tastefully decorated with palms, and a large audience was present. McAfee's Fifth Regiment Band furnished the music and contributed their full share to the enjoyment of the occasion.

---

DR. JAMES A. ETHRIDGE, a popular and well-known physician of Macon, died March 14th from the effects of chloroform. Dr. Ethridge was about to undergo an operation for fistula, and almost at the first inhalation of the chloroform his pulse and respiration ceased, and every effort to resuscitate him failed. Upon a preliminary examination of Dr. Ethridge no contraindication to the use of chloroform could be discovered. The physicians in attendance were Drs. Jackson, Little and Weaver. Dr. Ethridge was about forty years of age, and had practiced his profession successfully in Macon for many years. He was skilled in medicine and surgery, and also possessed of broad general information and intelligence. He was a member of the Board of Health of Macon, visiting physician to the city hospital and surgeon to the Georgia Southern and Florida Railroad. Dr. Ethridge's death was deeply mourned by a wide circle of friends.

---

OUR highly esteemed contemporary, the *Alabama Medical and Surgical Age*, will please accept our best thanks for the following kind words: "Our good neighbor, the ATLANTA MEDICAL AND SURGICAL JOURNAL, begins its fourteenth volume under the new series with this (March) issue. This journal is ably edited, neatly printed, and has been for these many years a welcome visitor to a large and appreciative list of the best doctors throughout this country. The "Old Atlanta" is true to the best interests of the profession, maintaining and upholding them at all times. As such, we hope she may live long and prosper."

The same to you, neighbor!

